# Altered Neural Activity Patterns in Amnestic MCI during Object‐Location Associations

**DOI:** 10.1002/alz70856_104046

**Published:** 2025-12-24

**Authors:** Jinxian Deng, Boxin Sun, Norman Scheel, David C Zhu, Benjamin M. Hampstead, Tongtong Li

**Affiliations:** ^1^ Michigan Alzheimer's Disease Research Center, Ann Arbor, MI, USA; ^2^ Michigan State University, East Lansing, MI, USA; ^3^ Albert Einstein College of Medicine, Bronx, NY, USA; ^4^ University of Michigan, Ann Arbor, MI, USA; ^5^ VA Ann Arbor Healthcare System, Ann Arbor, MI, USA

## Abstract

**Background:**

Existing work suggests that localized neural activity during a cognitive task reflects the effort level of the neuronal populations when fulfilling the task. This study, based on functional magnetic resonance imaging (fMRI), investigated the differences in neural activity between normal cognition (NC) and amnestic mild cognitive impairment (aMCI) individuals during object‐location associations (OLAs).

**Method:**

We used an event‐related fMRI paradigm to compare NC participants and aMCI patients as they encoded 90 ecologically relevant OLAs. A total of 43 right‐handed participants (19 NC, 24 aMCI) completed this study. The groups were well matched on demographics and brain volumetrics. The blood‐oxygen‐level‐dependent (BOLD) signals included both novel and repeated trials, where each trial lasted 14 seconds, corresponding to 8 sample points with the sampling period T = 2 s. We evaluated the neural activity of the regions in functional brain networks through the deconvolution of the hemodynamic response function (HRF) from the BOLD signals.

**Results:**

In most of the regions in the right executive control network (RECN), aMCI exhibited weakened neural activity than NC in novel trials. Concurrently, the differences between NC and aMCI were more significant in the novel trials. At the same time, the NC group exhibited lower neural activity in repeated trials, indicating that NC gained increased task‐relative information processing capacity and hence required less effort level to fulfill the same task post‐training.

The time‐varying neural activity in the RECN regions and Visuospatial regions were taken as primitive features, out of which NC and aMCI were found to be significantly different (i.e., *p*‐value < 0.05) in 15 of them. Taking the five features with the lowest *p*‐values as the input to a linear Support Vector Machine (SVM) model, an accuracy of 86% was achieved in NC and aMCI classification.

**Conclusion:**

aMCI exhibited significantly lower neural activity than NC in the RECN regions during OLAs, especially in novel trials. Overall, time‐varying neural activity in the OLA tasks was shown to be a promising biomarker for NC and aMCI classification.

**Funding**: NSF‐2032709/Li; NIH‐R35AG072262/Hampstead; NIH‐P30AG072931/Paulson; NIH‐P30AG024824/Yung.

